# A Manual of Medical Treatment or Clinical Therapeutics

**Published:** 1894-06

**Authors:** 


*A Manual of Medical Treatment or Clinical Therapeutics.* By I. Burney Yeo, M.D. Two Vols. Pp. 631 and 744. London: Cassell and Company, Limited. 1893.

The author gives a short account of the leading features, symptoms and pathological changes of each disease, and formulates the indications for treatment. Then follows the account of the treatment which should be adopted. This plan commends itself as the most suitable one for dealing intelligently with disease, and for avoiding the administration of drugs without sufficient reason. The only criticism we have to offer on the mode in which this plan is carried out is that, from the necessity of bringing the book within reasonable bounds and of not impairing its value for ready reference, the account of symptoms is often very brief, and thus loses much of its value. We are disposed to think that it would be sufficient to give fully the indications for treatment. It will thus be seen that the book is no mere compendium of drugs, but a thorough and scientific study of treatment in all its branches of general, medicinal and dietetic management. When necessary, some account is given of the indications for and mode of carrying out such special forms of treatment as massage, electricity, and hydrotherapeutics.

We find here a very satisfactory guide to all forms of treatment, and the character of the advice given, taken as a whole, is worthy of much praise. The reader is not left to choose out of a number of methods recommended, but is told from which he may expect his patient to derive the most advantage. This is as it should be; when a man turns to a work of this kind he wants first some definite advice for the ordinary type of case, and then secondly to know what should be done in the more complicated or unusual cases.

Of course one cannot expect to agree with the author as to all details of treatment, on many questions of therapeutics there is just ground for difference of opinion; but on all main points we feel sure that the author is a trustworthy guide, and that his statements will be found accurate. We have put the book for some little time past to the practical test of referring to it for the treatment of cases as they arose in the ordinary course of practice, and in every case we were satisfied with the information supplied.

We feel that we can thus cordially recommend the book as a useful and practical one, based on sound scientific principles, well adapted to the needs of everyday work, and supplying a distinct want in medical literature. In the limits of this notice it is impossible to discuss the details of a book of this sort; but after a thorough perusal of it, we are sure that it will be found a most satisfactory and valuable work of reference, for which we are emboldened to prophesy a great success. It possesses that essential feature for a book of this kind, a good index; the print is clear and good, and the volumes are of handy size.

